# Differential Expression of Stem Cell Markers in Ocular Surface Squamous Neoplasia

**DOI:** 10.1371/journal.pone.0161800

**Published:** 2016-09-01

**Authors:** Dilip Kumar Mishra, Uppala Veena, Swathi Kaliki, Abhinav Reddy Kethiri, Virender S. Sangwan, Mohammed Hasnat Ali, Milind N. Naik, Vivek Singh

**Affiliations:** 1 Ophthalmic Pathology Laboratory, L V Prasad Eye Institute, Hyderabad, Telangana, India; 2 The Operation Eyesight Universal Institute for Eye cancer, L V Prasad Eye Institute, Hyderabad, Telangana, India; 3 Champalimaud Translational Centre for Eye Research and Tej Kohli Cornea Institute, L V Prasad Eye Institute, Hyderabad, Telangana, India; 4 Prof. Brien Holden Eye Research Center, and Center for Ocular Regeneration, L V Prasad Eye Institute, Hyderabad, Telangana, India; 5 Clinical Epidemiology and Bio-Statistics, L V Prasad Eye Institute, Hyderabad, Telangana, India; 6 Manipal University, Manipal, Karnataka, India; National Institute of technology Rourkela, INDIA

## Abstract

Ocular Surface Squamous Neoplasm (OSSN) is the neoplasia arising from the conjunctiva, cornea and limbus. OSSN ranges from mild, moderate, severe dysplasia, carcinoma in situ (CIS) to squamous cell carcinoma (SCC). Recent findings on cancer stem cells theory indicate that population of stem-like cell as in neoplasia determines its heterogeneity and complexity leading to varying tumor development of metastatic behavior and recurrence. Cancer stem cell markers are not much explored in the cases of OSSN. In the present study, we aim to evaluate the expression of stem cells using stem cell markers mainly p63, ABCG2, c-KIT (CD117) and CD44 in OSSN tissue, which could have prognostic significance. The present study tries for the first time to explore expression of these stem markers in the cases of OSSN. These cases are subdivided into two groups. One group comprises of carcinoma in situ (n = 6) and the second group comprises of invasive carcinoma (n = 6). The mean age at presentation was 52 years; with 53 years for CIS group and 52 years for SCC group. From each group section from the paraffin block were taken for the IHC staining of p63, c-Kit, ABCG2 and CD44. Our experiments show high expression of P63 and CD44 in the cases of CIN and SCC. Both CIS and SCC displayed positive staining with p63, with more than 80% cells staining positive. However minimal expression of c-kit in both CIN and SCC. But surprisingly we got high expression of ABCG2 in cases of carcinoma in situ as compared to that of invasive squamous cell carcinoma. More than 50% of cells showed CD44 positivity in both CIS and SCC groups. Our results show for the first time that these four stem cells especially the limbal epithelium stem cells play a vital role in the genesis of OSSN but we need to explore more cases before establishing its clinical and biological significance.

## Introduction

Ocular surface squamous neoplasia (OSSN) is the most common tumor of the ocular surface with an estimated incidence 0.02 to 3.5 cases per 100000 worldwide [1]. It is known to be the third most common tumor of eye after retinoblastoma and melanoma [2]. OSSN is strongly associated with ultra violet radiation exposure, Human Immunodeficiency Virus, Human papilloma virus infection, chronic use of contact lens, exposure to petroleum product drugs, smoking, and other unknown factors[1,3]. The diagnosis of OSSN is mainly by histopathological analysis. The OSSN ranges from mild, moderate, severe dysplasia, carcinoma in situ (CIS) to squamous cell carcinoma (SCC) [4,5]. Reported recurrence rate of OSSN is 15–52%. Lee and Herst (1997) have reported a 17% recurrence after excision of conjunctival dysplasia, 30% for SCC of conjunctiva and 40% after excision of CIS [6]. However, OSSN is considered as a low-grade malignancy. In a recent study of 64 cases with OSSN by Chauhan et al., 18% cases with squamous cell carcinoma developed metastasis and 12% died[7], as compared to earlier reports of metastasis varying from 0 to 8%[8]. Similarly, many groups did not support an association between histopathological grade and recurrence [9,10]. This changing trend of increasing metastasis leads us to study and explore the role of unexplored factors like cancer stem cells in OSSN.

Recent findings on cancer stem cells theory[11], indicates that population of stem-like cell as in neoplasia, determines its heterogeneity and complexity leading to varying tumor development of metastatic behavior and recurrence[12]. Cancer stem cell expression were discovered in plethora of human malignancies; like CD133, CD44 and ALDH1 in solid tumors, CD34 in hematological malignancies[13]. p63, one of the epithelial stem cell marker is preferentially shown to be expressed in variety of neoplasms including SCC, cervix, prostate, urothelium, endometrial and thyroid carcinoma[14]. Higher expression of c-Kit (CD117) in oesophageal. SCC is shown to be associated with poor survival and may be used as a prognostic marker [15].ABCG2 one of the known stem cell marker is an efflux transporter and is involved in multidrug resistance. It has been shown to be regulated by MAPK pathways in cancer progression like laryngeal SCC[16]. In our recent study we have also shown ABCB2 and p63 alpha as one of the prominent stem cell marker from limbus, [17] which may be involved in the origin of OSSN.

To our knowledge, till date, there are very few studies reporting the expression cancer stem cells in OSSN. In the present study, we aim to evaluate the expression of stem cells using stem cell markers mainly p63, ABCG2, c-KIT (CD117) and CD44 in OSSN tissue, which could have prognostic significance.

## Methods

### OSSN sample details

The study were approved by the Institution review board, Hyderabad Eye Research Foundation, LV Prasad Eye Institute, Hyderabad, India[IRB No: LEC-04-15-043]. Study was in accordance with the tenants set forth in the declaration of Helsinki for research. Written informed consent from the donor or the next of kin was obtained for the use of these samples in research. All consent form are kept and documented in pathology department for any future need. After excision biopsy and histopathology processing of OSSN, the tissues of CIS (n = 6) and SCC cases (n = 6) were obtained from the pathology laboratory. The clinic pathological details of the CIS and SCC patients are listed in Tables 1 and 2 respectively.

**Table 1:** Clinicopathological features of six patients with carcinoma-in-situ (CIS).

S.No	Age/Sex	Laterality	Occupation	Quadrantic tumor location	Histopathology Diagnosis	Resected margins
1	81/M	OD	House wife	Nasal	CIS, the tumor extends focally to the base and the corneal aspect of the biopsy	Free of tumor
2	41/M	OD	Contractor	Temporal	CIS	Free of tumor
3	74/F	OD	House wife	Temporal	Actinic keratosis associated with CIS	Super lateral margin shows mild focal dysplasia.
4	62/M	OS	Business	Temporal	CIS	Superior margin shows mild focal dysplasia.
5	25/M	OD	Agriculture	Temporal	CIS with area of micro invasion associated with actinic keratosis	Superior margin shows mild focal dysplasia.
6	35/M	OS	Business	Temporal	Moderate dysplasia to CIS associated with actinic keratosis	Free of tumor

**CIS:** carcinoma in situ, **OD:** left eye, **OS:** right eye, **M:** male, **F:** female

**Table 2:** Clinicopathological features of six cases with invasive squamous cell carcinoma (SCC).

S.No	Age/sex	Laterality	Occupation	Quadrantic tumor location	Histopathology diagnosis	Resected margins
1.	61/M	OD	Business	Temporal	SCC, moderately differentiated	Free of tumor
2.	70/F	OS	Business	Nasal	SCC, deeper margin is close but free of tumor	Free of tumor
3.	40/M	OD	Business	Temporal	Invasive SCC, with associated carcinoma in situ	Free of tumor
4.	57/M	OS	Farmer	Temporal	Well differentiated SCC	Free of tumor
5.	19/M	OS	Student	Nasal	Poorly differentiated SCC with CIS and dysplastic changes in the adjacent epithelium.	Free of tumor
6.	63/M	OD	Business	Temporal	Poorly differentiated SCC	Free of tumor

**SCC:** squamous cell carcinoma, **OD:** left eye, **OS:** right eye, **M:** male, **F:** female

### Sample collection and processing

OSSN tissue biopsy samples were fixed in 10% neutral-buffered formalin (catalogue no-24008, Fisher Scientific, India) for 24 hours after excision, grossed and representative area of tumor and margins were sectioned. These sectioned tissues were again processed in automatic tissue processor and embedded in paraffin wax (catalogue no-64271, Merck KGaA, USA). The paraffin embedded tissue blocks were cut into 4μm thickness using microtome (Leica, RM2125RTS) and were further processed for haematoxylin-eosin (H&E) staining and immunohistochemistry.

### H&E staining procedure

Paraffin sections of OSSN tissues samples were deparaffinized using serial solutions of xylene(catalogue no-35417, Thermo Fisher Scientific India Pvt Ltd, Mumbai, India)and hydrated through serial dilutions of 100%, 90%, 80% alcohol (catalogue no-26897, Thermo Fisher Scientific India Pvt Ltd, Mumbai, India). Then washed with running tap water and stained with Harris’s hematoxylin (catalogue no-H3136100G, Sigma aldrich, USA) for 10minutes, and washed with running tap water for 5minutes. They were further differentiated using 1% acid alcohol, washed with running tap water for 10 minutes, counter stained with1% Eosin Y (catalogue no-E4382, Sigma-aldrich, Germany) for 1 to 2 minutes, dehydrated with 80%, 90%, 100% alcohols and cleared with xylene, and mounted with DPX mount medium (catalogue no-46029 Fine-chem limited, Mumbai, India)[17].

### Immunohistochemistry

Deparafinization of all 4μm thick paraffin sections of OSSN tissue samples were done with xylene and rehydrated through graded alcohol of 100%, 90%, 80% dilution. This procedure was followed by blocking with endogenous peroxidase activity by using methanol (catalogue no-39192-L25 Fine-chem limited, Mumbai, India) containing 3% H_2_O_2_ (catalogue no-18706, Thermo Fisher Scientific India Pvt Ltd, Mumbai, India) for 20 minutes incubation and then washed with phosphate buffer saline (PBS) for 3 times. After that antigen retrieval by heating the sections with EDTA buffer (pH-9.0) for 30 minutes The antigen retrieval slides were incubated with bovine serum albumin (catalogue no-A7906, Sigma, USA) for 20 minutes to avoid nonspecific binding of antibody. They were then incubated with primary antibodies (Table 3) and washed three times with PBS and further incubated with secondary antibodies for 30 minutes. They were then washed thrice with PBS, incubated for 5 minutes with DAB substrate (catalogue no-HK124-9K, BioGenex, CA, USA), washed with distilled water, and counter stained with Harris’s hematoxylin. Finally, the slides were mounted with cover slips using a DPX (catalogue no-46029 L02s d Fine-chem limited, Mumbai, India,) mounting medium.

**Table 3:** Details of antibodies used in our study.

Antibody	Dilution of antibody	Incubation time	Catalogue number
p63	As per kit	1 hour at room temperature	416-5M, BIOGENEX
c-Kit (CD117)	1:300	1hour at room temperature	4502, Dako
ABCG2	1:10	1hour at room temperature	3380, Abcam
CD44	1:100	1hour at room temperature	51037, Abcam
Secondary antibody	As per kit	Half an hour at room temperature	HK595-YAK, BIOGENEX

### Imaging and quantification

Specimens were examined with an Olympus light microscope (BX51), and quantitative analysis of markers were done by counting 1000 tumor cells in 10X high power magnification in 3 randomly selected fields of each slide. The counting was done in real time and not in the photographic images. Quantification was done, based on the total average number of positive cells stained from each set of 3 randomly analyzed fields. Two independent investigators confirmed the quantification and histopathological analysis of each slide. Immunohistochemical analyses for all samples were repeated at least 3 times for each case.

### Statistical analysis

All statistical analyses were performed using the R software (version 2.12). ANOVA test was used to test the mean difference between CIS and SCC group. A multiple comparisons of means by Tukey contrasts were used to test the variation within each group of CIS and SCC.

## Results

### Clinical Presentation

**Fig 1:**
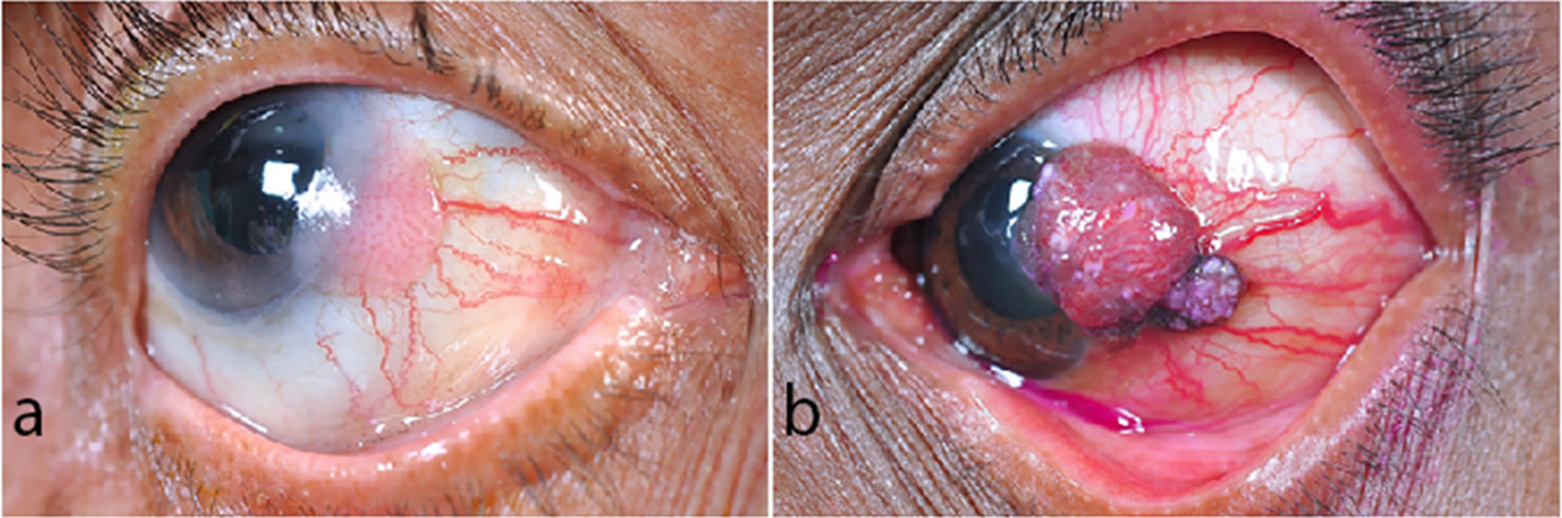
Clinical presentation of ocular surface squamous neoplasia (OSSN). **(a)** A 74 year old female with OSSN involving nasal peripheral cornea, limbus, and bulbar conjunctiva. The lesion was carcinoma-in-situ on histopathology. **(b)** A 56 year old female with partially pigmented OSSN involving temporal peripheral cornea, limbus, and bulbar conjunctiva. The lesion was invasive squamous cell carcinoma on histopathology.

A total of 12 patients were included in the study. The mean age at presentation was 52 years, with 53 years for CIS group and 52 years for SCC group. There were 10 males and 2 females. The most common presenting complains were leukoplakic lesion in CIS and papillomatous growth in SCC. The tumor was located in the temporal quadrant in nine of the 12 cases. The representative images of Clinical presentation of ocular surface squamous neoplasia is shown in Fig 1.

### Histopathological analysis

**Fig 2:**
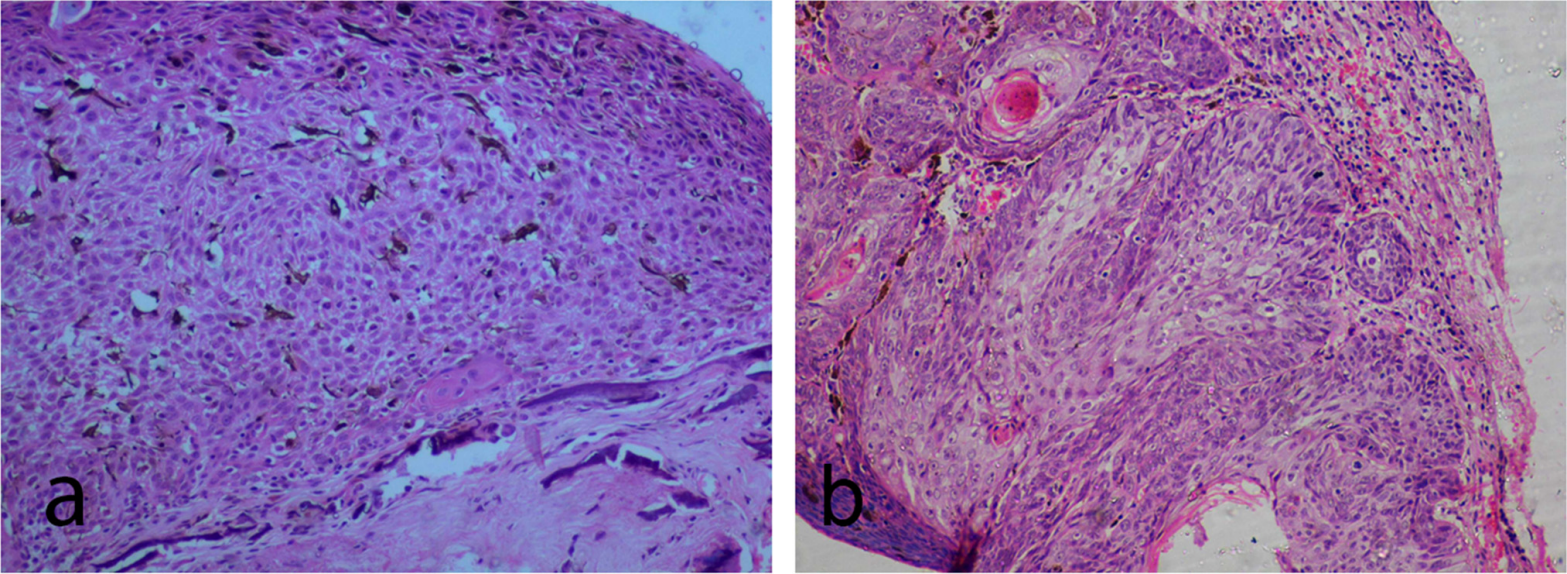
Histopathology of ocular surface squamous neoplasia (OSSN). **(a)** Photomicrograph (Hematoxylin and eosin stain, 10x magnification) of carcinoma in situ section showing acanthotic squamous epithelium displaying full thickness dysplasia. Dysplastic cells show loss of polarity with hyperchromatic pleomorphic nuclei, vesicular chromatin, and small nucleoli. **(b)** Photomicrograph (Hematoxylin and eosin stain, 10x magnification) of squamous cell carcinoma showing dysplastic squamous epithelial cells arranged in cords, tubules and solid sheets, and lamellated keratin pearl. These dysplastic squamous epithelial cells have breached the basement membrane and are infiltrating into the stroma.

Six patients had CIS and 6 had SCC on histopathology. Microscopy of the carcinoma in situ shows edacanthotic squamous epithelium displaying full thickness dysplasia in one case. The dysplastic cells have hyperchromatic, pleomorphic nuclei, vesicular chromatin, and small nucleoli. 1 to 2 mitotic figures per 10 high power fields (HPF) were noted (Fig 2A). Basement membrane was intact in CIS cases. Invasive SCC cases showed full thickness dysplasia with breach in the continuity of basement membrane and infiltration of tumor cells into the substantia propria (stroma) in the form of sheets, tubules and cords (Fig 2B). Of the six cases, 2 cases showed well-differentiated acellular keratin pearl formation. Substantia propria (stroma) in SCC showed dense chronic inflammatory cells, hemorrhage and dilated blood vessels.

### Expression of p63 in CIS and SCC

**Fig 3:**
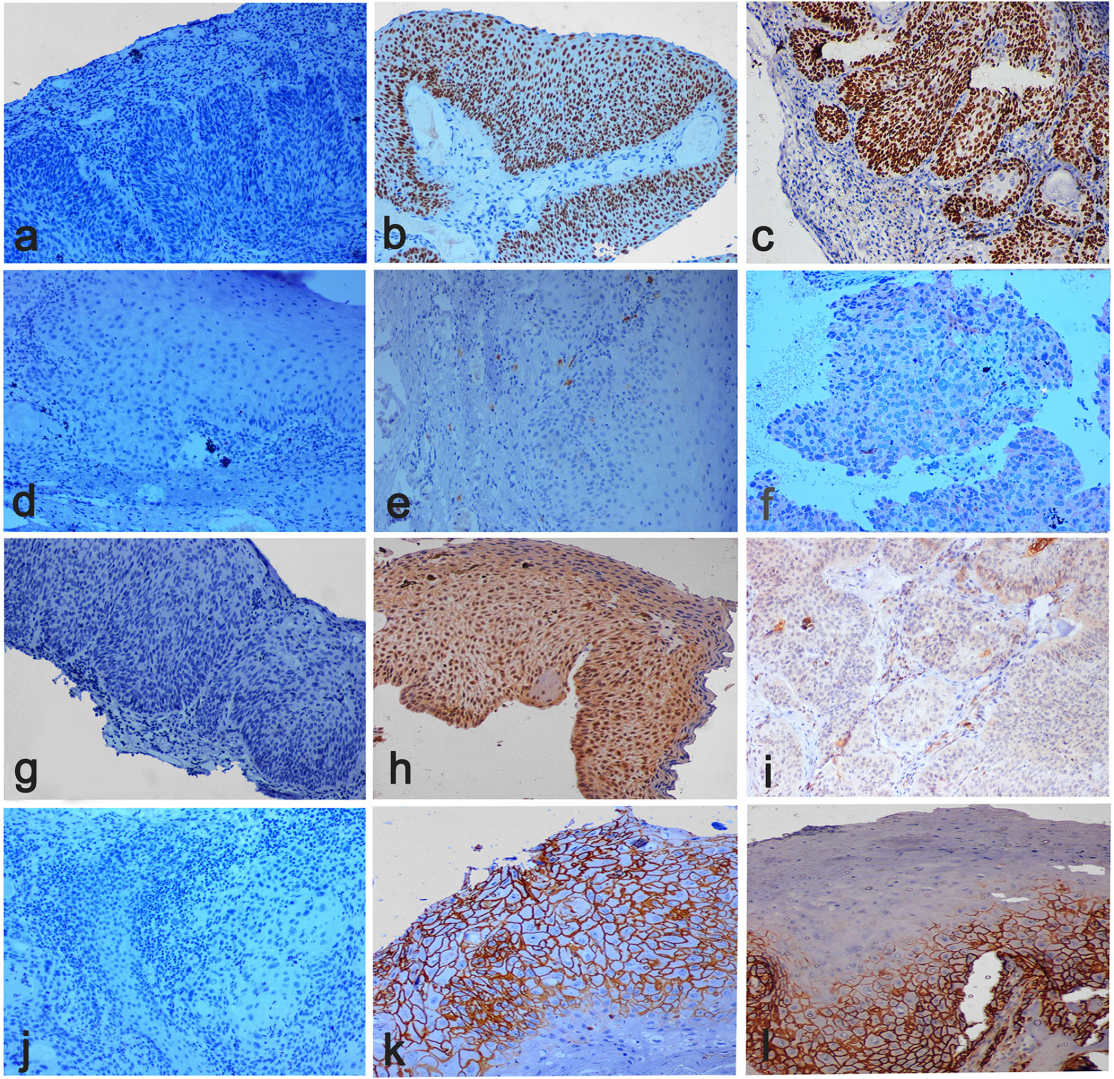
Stem cell markers for ocular surface squamous neoplasia (OSSN). **a)** Negative staining of p63 marker. **b)** Overexpression of p63 in the nucleus of neoplastic cells in the epithelium in a case with CIS. **c)** Over expression of p63 in nucleus of neoplastic cells in the epithelium and substantia propria (stroma) in a case of invasive squamous cell carcinoma (SCC). **d)** Negative staining of c-Kit (CD117) marker. **e)** Expression of c-Kit (CD117) in the cytoplasm of a few neoplastic cellsin the epithelium in a case of CIS. **f)** Expression of c-Kit (CD117) is in the cytoplasm of a few neoplastic cells in the epithelium and stroma in a case of SCC. **g)** Negative staining of ABCG2 marker. **h)** Expression of ABCG2 in the nuclear membrane of neoplastic cells in the epithelium in a case of CIS. **i)** Expression of ABCG2 in the nuclear membrane of neoplastic cells in the epithelium stroma in a case of SCC. **j)** Negative staining of CD44 marker. **k)**High expression of CD44 in the cytoplasmic membrane of neoplastic cells in the epithelium in a case of CIS. **l)** High expression of CD44 in the cytoplasmic membrane of neoplastic cells in the epithelium and stroma in a case of SCC. All figures were taken at 10X magnification.

**Fig 4:**
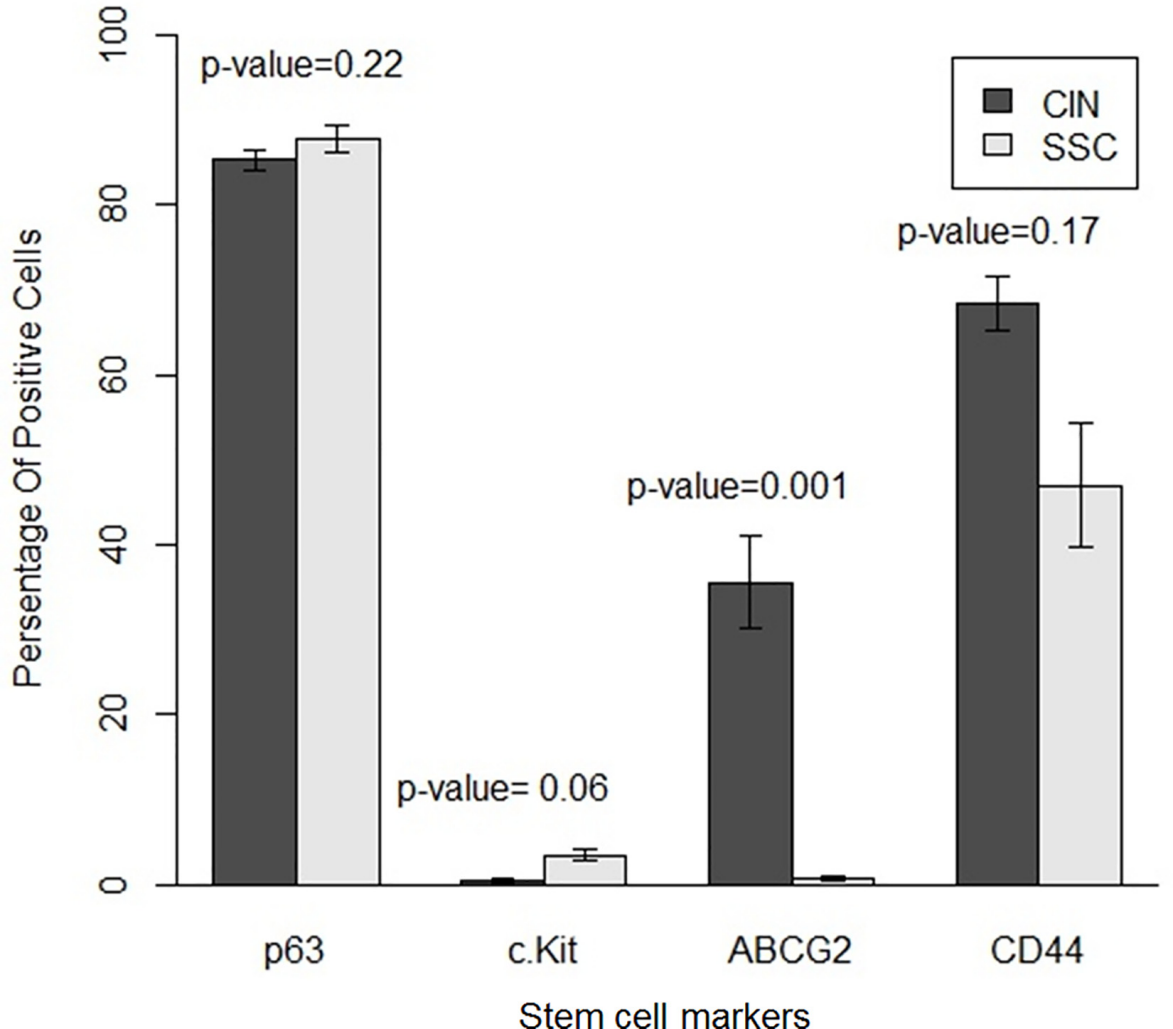
Quantitative analysis of stem cell markers for p63, c-Kit, ABCG2, CD44 in carcinoma-in-situ (CIS) and squamous cell carcinoma (SCC) by using ANOVA test.

**Fig 5:**
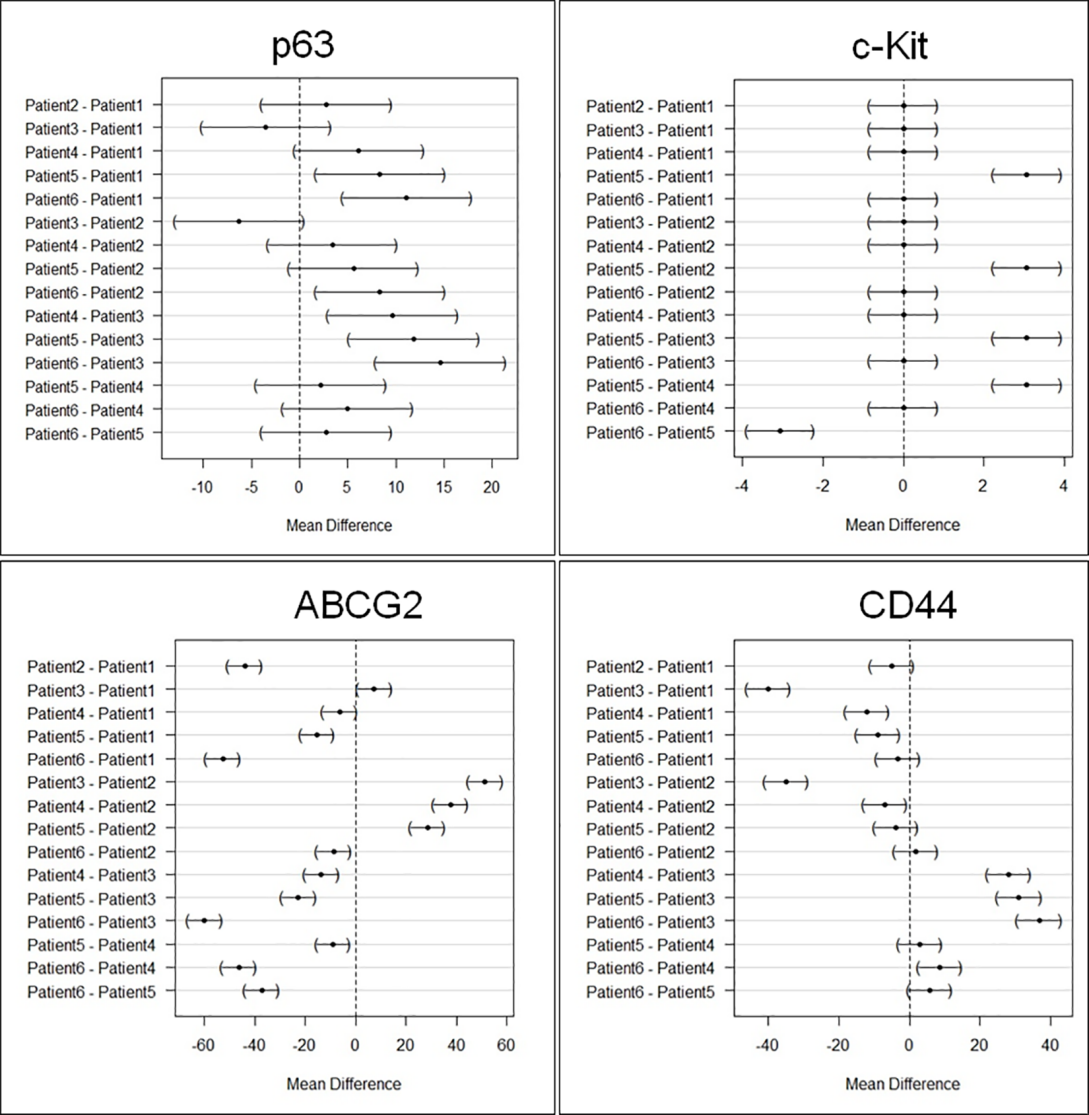
Intra-group variation in the stem cell marker expression in carcinoma-in-situ cases by using Tukey contrast.

**Fig 6:**
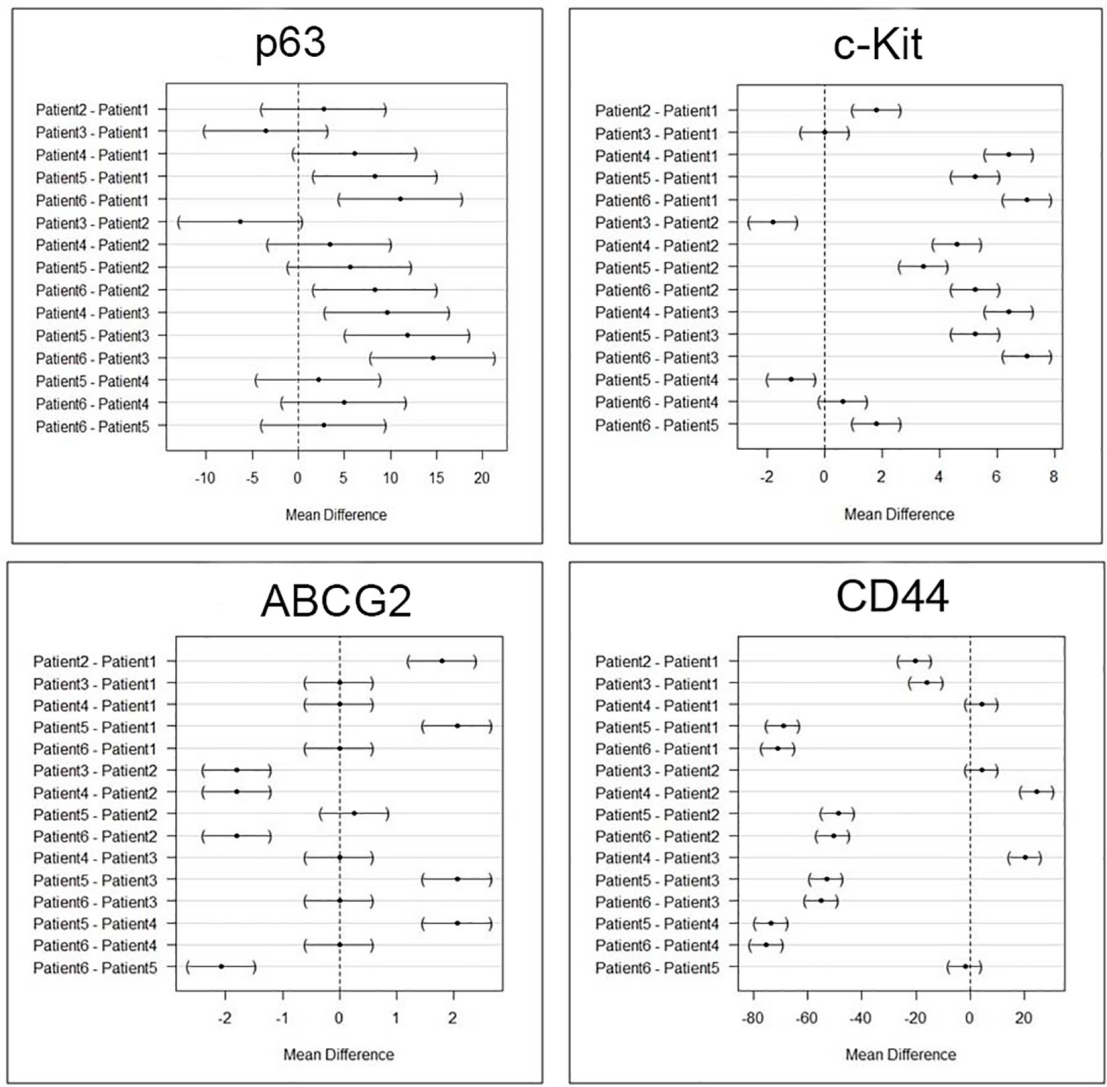
Intra-group variation in the stem cell marker expression in invasive squamous cell carcinoma cases by using Tukey contrast.

Both CIS and SCC displayed positive staining with p63, with more than 80% cells staining positive (Fig 3B and 3C). However quantitative expression of p63 was higher in SCC compared to CIS (Fig 4), but was not statistically significant [p-value 0.22]. We also analyzed variation within SCC group as well as CIS group. There was variation within each group but was not statistically significant in both CIS and SCC, as shown in mean difference plot for p63 marker in Figs 5 and 6.

### Expression of c-Kit [CD117] in CIS and SCC

Our results suggest some positive expression of c-Kit in SCC cases but very low expression in CIS as shown in the Fig 3E and 3F and Fig 4. However, quantitative difference in expression of c-Kit in both the groups was not significant (p ≥ 0.06). We also analyzed variation within SCC group as well as CIS group. There was variation within each group but was not statistically significant in both CIS and SCC, as shown in mean difference plot for c-Kit marker (Figs 5 and 6).

### Expression of ABCG2 in CIS and SCC

There was positive expression of ABCG2 in CIS but very less positive expression in SCC (Fig 3H and 3I and Fig 4). However expression of ABCG2 was significantly higher in CIS as compared to SCC cases (p ≥ 0.001). There was variation within each group but was not statistically significant in both CIS and SCC, as shown in Figs 5 and 6 in mean difference plot.

### Expression of CD44 in CIS and SCC

More than 50% of cells showed CD44 positivity in both CIS and SCC groups (Fig 3K and 3L). However, expression of CD44 was higher in CIS cases as compared to SCC (Fig 5). We also analyzed variation within CIS group as well as SCC group. There was variation within each group but was not statistically significant in both CIS and SCC, as shown in Figs 5 and 6.

## Discussion

Targeted cancer stem cell therapy is an evolving treatment strategy for various cancers. Our current study shows the presence of stem cells in OSSN using known stem cell markers mainly p63, c-Kit, ABCG2, CD44 with differential expression in CIS and SCC cases. Based on the current literature, these stem cell markers have never been reported earlier in OSSN. However, a recent study has shown the elevated expression of ABCB5 in nine OSSN cases [18]. ABCB5 has already been demonstrated in chemo resistant stem cells, as well as drug efflux transportation of doxorubicin in human melanoma[19].

Limbal basal epithelium mainly contains the stem cell population[20]. Earlier study by Chui J et al has shown that stem cells arrange in micro clusters in the basal epithelium in 12% pterygium cases[21]. Putative markers like ABCB5[22], ABCG2, and p63[23], aid in the recognition of limbal epithelial basal stem cells. In our study, we observed expression of p63 and ABCG2, which indicates that the origin of OSSN is from the limbal stem cells. Nevertheless, it is still debatable due to the lacunae in our knowledge of specific stem cells present in conjunctiva, limbus or cornea.

Histopathology is the gold standard in the diagnoses of various grades and types of OSSN, and gives a fair understanding of disease prognosis[24]. In an earlier study from our group, it was noted that HIV-positive individuals have poor ocular prognosis with higher need for extended enucleation, exenteration, or both, however the significance of stem cells were not explored in this study[25]. In the cases of skin cancer, p63 are expressed at higher rate [26,27]. Similarly, in our study there was higher expression of p63, in both CIS and SCC. p63 has been shown to increase the expression of tumor promoting genes/factors like CD44, COX2, and β catenin[28]. p63 also promotes the expression of keratin thus may lead to epithelial proliferation, differentiation and progression of OSSN. (ABCG2 is a known putative stem cell marker as well as a chemo-resistant marker[29]. In our study, ABCG2 expression was low in SCC compared to CIS (Fig 4). c-Kit [CD117] is also one of the marker for identification and characterization of hematopoietic stem progenitor cells[30], which showed low expression in both CIS and SCC in our study (Fig 5).

**Fig 7:**
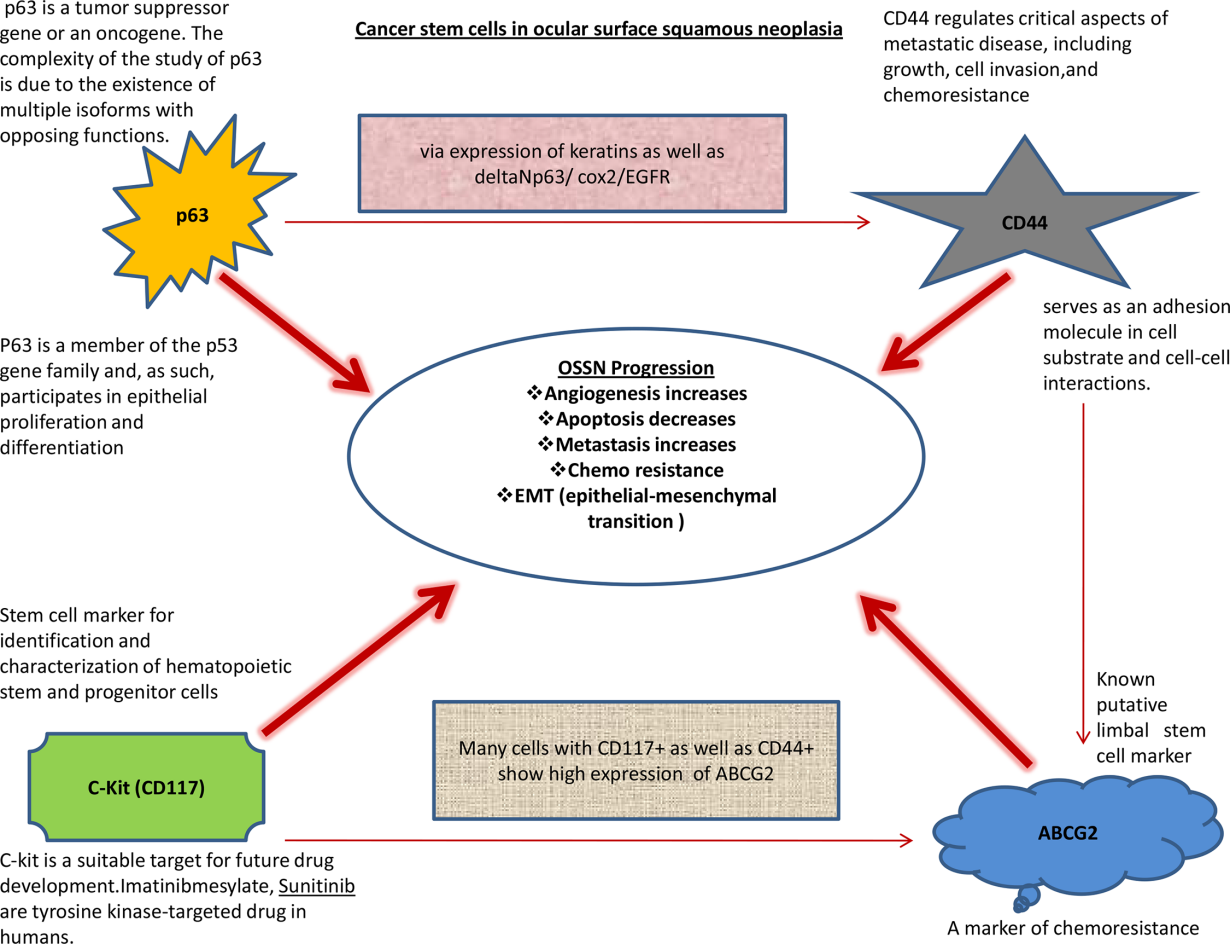
Our hypothesis regarding the role of stem cell markers in OSSN. This figure shows the expression of stem cell markers (p63, CD44, c-Kit and ABCG2) in OSSN and how these makers might be playing role in the progression of OSSN and may have role in better prognosis at late stage of neoplasia.The figure also correlates the interaction between these stem cell markers (from the various published studies) and there probable role in OSSN (15,16,27,30).

We hypothesize that with the progression of OSSN, there is high expression of p63 as well as c-Kit [CD117], which in turn may regulate the expression of CD44 and ABCG2 (Fig 7). p63 is known to promote the expression of CD44 via expression of keratins as well as ΔNp63/ COX2/ EGFR [28]. Similarly c-Kit has been shown to increase the expression of ABCG2 in c-Kit and CD44 positive cells[31]. In the present study, we also hypothesize that stem cells expressing p63, c-Kit, ABCG2, and CD44 have a role in the progression of OSSN (Fig 7), and needs further studies to confirm the same.

Studies have shown in breast and other cancers that CD44 contribute to its progression, correlates with advanced grade tumors and offer a potential prognostic and therapeutic target in such tumors[32]. Our results are too concurrent with higher expression of CD44 both in CIS and SCC cases of OSSN (Figs 4 and 7).

Similarly, the c-kit (CD117) expression is a feature of more aggressive retinoblastoma and these c-Kit pathway has been shown to be involved in pathological processes, including hematopoiesis, oncogenesis, etc[33,34]. A recent finding has shown that c-kit expression is a valuable predictor of prognosis and survival, especially in thick (> 4 mm) melanoma[35]. We think that c-Kit, however low in expression (Fig 4), may be the initial trigger in the process of OSSN progression (Fig 7) and it has been reported that c-Kit can trigger the higher expression of ABCG2, a known limbal stem cell marker. Elevated expression of ABCB2 may be involved in the pathogenesis of OSSN. Our result also demonstrates that c-Kit is expressed in both categories of OSSN but higher in SCC cases, however these % are low. Nevertheless, we need to consider that percentage of cancer stem cells or tumor-initiating cells are generally less than 5% of total volume of any tumor mass reported till date[36] and even these low % of stem cell are capable enough to change the tumor progression pathways, prognosis of OSSN and finally the outcome of any cancer therapy. OSSN occurs predominantly in the limbal area where the limbal stem cell (LSC) niche resides, but the molecular mechanism and pathogenesis of OSSN are still unknown[37] and we attempt to extrapolate how these stem cells might be playing role in OSSN progression but these need to be further validated in more samples using different biological tools and techniques (Fig 7).

The limitation of our study is the limited number of OSSN samples. Nevertheless, its strength is that, it is the first to analyze stem cells markers for OSSN including CIS and SCC. In summary, our results have shown the existence of stem cells in OSSN using four stem cell markers (p63, ABCG2, c-Kit& CDD44). Studies have shown that minimum amount of cancer stem cells can lead to disease relapse, and removal of cancer stem cells would mean the eradication of the disease. However, the major hurdle is to find treatments that can kill these specific stem cells without harming the normal cells[38]. The screening of cancer stem cells can be used to prognosticate the severity of OSSN and possibly its metastatic potential. A prospective study involving a large number of patients is needed to study the role of these markers in tumor differentiation into SCC and CIS, and its prognostic value.
